# A heart in a cage: cardiac compression and cardiovascular manifestations of pectus excavatum—a case report

**DOI:** 10.1093/ehjcr/ytag682

**Published:** 2026-09-15

**Authors:** Carlos Salazar, Jorge Tolosa-Cubillos, Victor Benenati, German Gomez

**Affiliations:** Clínica de Marly, Calle 50 No. 9-67, Bogotá, D.C. 110231, Colombia; Clínica de Marly, Calle 50 No. 9-67, Bogotá, D.C. 110231, Colombia; Clínica de Marly, Calle 50 No. 9-67, Bogotá, D.C. 110231, Colombia; Clínica de Marly, Calle 50 No. 9-67, Bogotá, D.C. 110231, Colombia

**Keywords:** Pectus excavatum, Right ventricular compression, Thoracic surgery

## Summary

We present the case of a 40-year-old woman with a background history of endometriosis and neurocardiogenic syncope of the vasodepressor subtype. During the course of her cardiac rehabilitation programme, a change in the clinical presentation of her syncopal episodes was observed, with the new onset of cardiovascular chest pain occurring subsequent to haemodynamic collapse. Multimodal cardiac imaging revealed significant right ventricular compression attributable to pectus excavatum. Surgical correction was subsequently undertaken, resulting in complete clinical recovery, resolution of right ventricular compression, and the absence of syncopal episodes over a two-year follow-up period.This case highlights the importance of a thorough clinical assessment combined with the judicious use of multimodal imaging in achieving an accurate diagnosis and formulating an appropriate therapeutic strategy. Ultimately, this approach led to the complete resolution of symptoms and a favourable impact on long-term survival.

## Case description

We present the case of a 40-year-old woman with neurocardiogenic syncope of the vasodepressor subtype, occurring at rest with identifiable triggers and an initially benign profile, managed with lifestyle modification and structured cardiac rehabilitation.

Over the following 2 years, syncopal episodes persisted but their presentation evolved: episodes began occurring during exertion, and on regaining consciousness, the patient reported anginal chest pain with symptomatic hypotension, prompting cardiovascular re-evaluation.

Cardiac magnetic resonance imaging demonstrated a 13 mm pectus excavatum causing mild compression at the right atrioventricular junction. Coronary computed tomography angiography confirmed the deformity, with the inferior sternum directly apposed to the anterior cardiac surface; the right coronary artery was unaffected (*Figure 1*).

**Figure 1:**
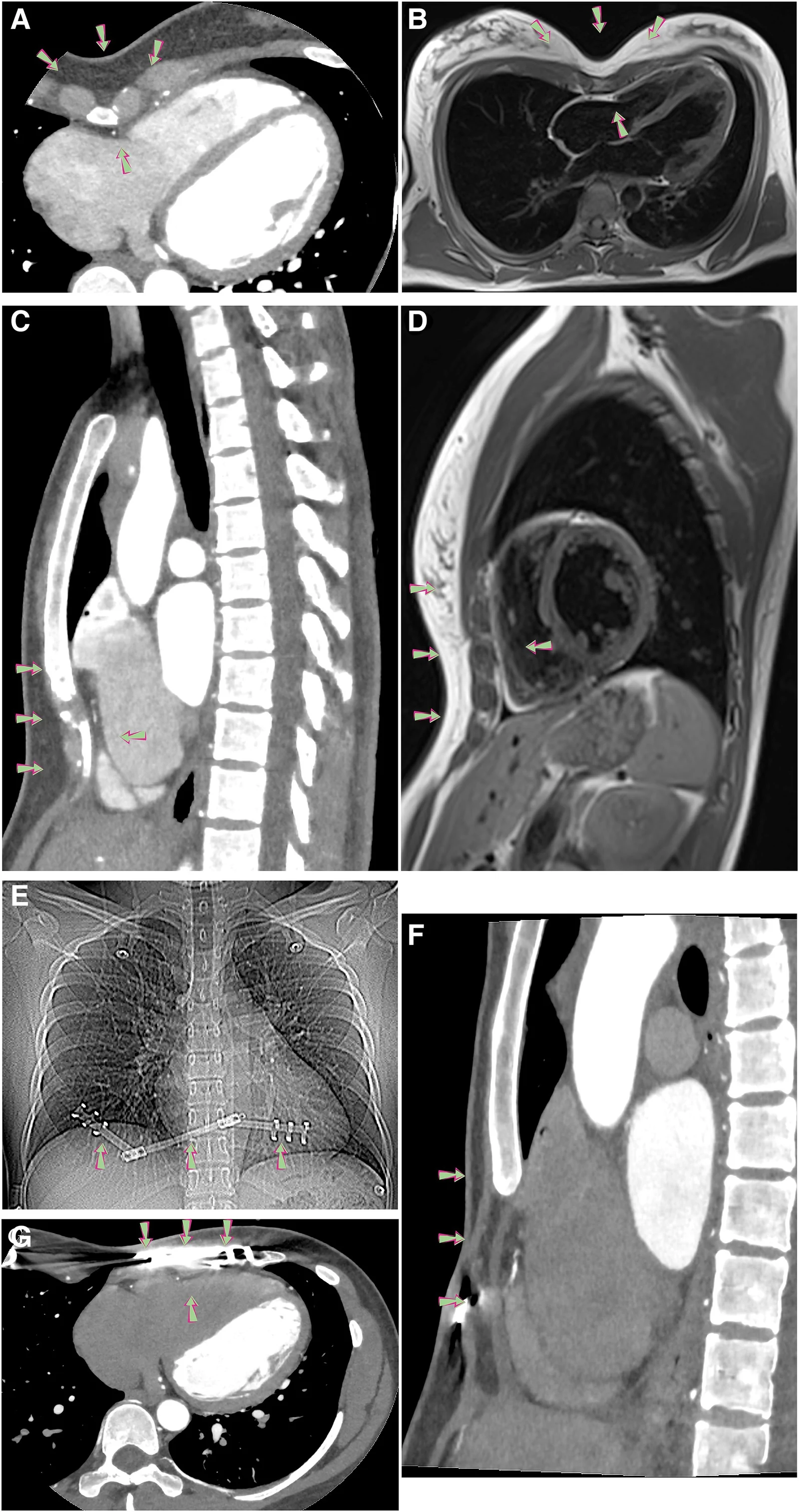
Computed tomography and magnetic resonance imaging images showing right ventricular compression from pectus excavatum before surgery, and its resolution after sternal repair. (*A*) Coronary computed tomography angiography (axial view) demonstrating sternal compression of the coronary sulcus and the anterior wall of the right ventricle due to pectus excavatum. (*B*) Cardiac magnetic resonance imaging (T_1_-weighted, four-chamber view) showing compression of the anterior cardiac wall secondary to pectus excavatum; no further abnormalities were identified using this imaging modality. (*C*) Coronary computed tomography angiography (sagittal view) illustrating inferior pectus excavatum with resulting compression of the anterior cardiac wall. (*D*) Cardiac magnetic resonance imaging (T_1_-weighted, short-axis view) demonstrating compression and compromise of the right ventricle due to pectus excavatum. (*E*) Scout view demonstrating osteosynthesis material following surgical repair via thoracotomy. (*F*) Post-procedural cardiac computed tomography angiography (sagittal view) showing cardiac decompression following the correction of inferior pectus excavatum. (*G*) Cardiac computed tomography angiography (axial view) demonstrating the sternal osteosynthesis material and resolution of the compression of the right ventricle.

The temporal relationship between the exertional symptoms and the imaging-confirmed cardiac compression, together with the mechanistic plausibility of impaired right ventricular filling during exertion, supported a causal role for pectus excavatum. Given objective evidence of atrioventricular junction compression correlating with symptoms, surgical sternal reconstruction was recommended and performed via thoracotomy.

Postoperatively, the patient completed further rehabilitation uneventfully and remained entirely asymptomatic at 2-year follow-up.

Pectus excavatum is the most common congenital anterior chest wall deformity, occurring in ∼1 in 300–400 live births.^1^ Although often regarded as cosmetic, it may cause significant haemodynamic effects through compression of cardiac structures, particularly the right ventricle and, less commonly, the right coronary artery.^1^ Most patients nonetheless follow a benign course, and mild ventricular dysfunction or pericardial effusion is a common incidental finding without prognostic significance.^4,5^ Correction should therefore be reserved for patients with objective cardiopulmonary compromise or compression-related symptoms; surgical repair yields significant post-operative improvement in cardiovascular parameters.^2,3^

In conclusion, this case illustrates an uncommon but clinically important cardiovascular manifestation of pectus excavatum. Although causality cannot be established from a single case, the temporal association between anatomical compression and symptom evolution, and its sustained resolution after surgery, supports this interpretation. The case highlights the value of multimodal imaging in diagnosis and of judicious surgical reconstruction. Clinicians should consider pectus excavatum as a cause of exertional cardiovascular symptoms in patients with an initially benign syncopal profile.

## Data Availability

All data relevant to this case report are included within the article. No additional datasets were generated or analysed in support of this case report.
